# Acute mastoiditis in an Italian pediatric tertiary medical center: a 15 – year retrospective study

**DOI:** 10.1186/s13052-018-0511-z

**Published:** 2018-06-18

**Authors:** Claudia Balsamo, Carlotta Biagi, Margherita Mancini, Ilaria Corsini, Rosalba Bergamaschi, Marcello Lanari

**Affiliations:** Pediatric Emergency Unit, Azienda Ospedaliera – Universitaria di Bologna, Policlinico Sant’Orsola – Malpighi, via Massarenti 10, 40100 Bologna, Italy

**Keywords:** Acute mastoiditis, Acute otitis media, Antibiotics

## Abstract

**Background:**

Acute mastoiditis is the main suppurative complication of acute otitis media. Its incidence ranges from 1.2 to 4.2/100.000 children/year and a rise has been reported in the last years. There are controversial data regarding risk factors for mastoiditis and its complications.

Aim of the study: to evaluate demographics and clinical characteristics of children with acute mastoiditis and to identify possible risk factors for complications.

**Methods:**

We retrospectively reviewed medical charts of all the children aged 1 month-14 years admitted to our Paediatric Emergency Department from January 2002 to December 2016.

**Results:**

One hundred forty-seven cases (97 males and 50 females) were included in the analysis, mean age was 4.8 ± 3.6 years and 28.2% of the patients were younger than 2 years. We found an increasing number of mastoiditis per year during the last 3 years of the study. Children younger than 2 years were less treated with antibiotics for acute otitis media or treated for a shorter period (*p* < 0.05), while they were treated at higher antibiotic’s dosage for mastoiditis (*p* < 0.01). Older children presented more frequently with symptoms such as earache or retroauricular pain (*p* < 0.0001, *p* < 0.001). We didn’t identify any risk factor for mastoiditis complications in our study.

**Conclusions:**

Despite the introduction of pneumococcal vaccines, the incidence of acute mastoiditis in our population has not been reduced during the last years. We have to face all the reasons why this condition is still relevant, such as antibiotic resistance, new pathogens involved and a possible role played by the implementations of therapeutic acute otitis media guidelines restricting the use of antibiotics in this disease. A particular attention should be given to younger children where signs and symptoms may be less pronounced, therefore acute otitis media or mastoiditis may be misunderstood and appropriate treatment delayed.

## Background

Acute mastoiditis (AM) is the most common severe complication of acute otitis media (AOM) and it is due to the extension of the infection to the mastoid process of the temporal bone [1].

After the introduction of the routine use of antibiotics in AOM in the 1950s, the percentage of progression from AOM to AM fell from 20 to 0.4% [2] and the number of admission due to AOM had halved [3, 4]. The incidence of AM has consequently declined but there is currently debate whether it is rising or not [5, 6]. Nowadays AM has an estimated incidence between 1.2 and 4.2/100.000 children/year [7]. Those authors, claiming an increase in the number of case of AM during the last years, refer this event to the selection of bacterial strains resistant to antibiotics [2, 8], secondary to the routine use of antibiotics and pneumococcal vaccines [1], and to the “watchful waiting” practice proposed by AOM guidelines [9].

In the late 1980s the traditional antibiotic treatment for AOM was accused of contributing to the diffusion of drug resistant pathogens, moreover AOM showed to have high spontaneous resolution rates [10]. Therefore many scientific societies proposed their guidelines about diagnosis and treatment for AOM, every author strongly recommended pain management and suggested an observational management in low risk patients consisting in deferring antibacterial treatment for 48–72 h, the so call “watchful waiting strategy” [9, 11].

Italian guidelines were published in 2010 in order to define better AOM criteria and recommendations for watchful waiting and antibiotics’ prescription [12]. In 2015 Emilia Romagna region updated its previous guidelines edited in 2007, these indications partially differs from the national recommendations [13]. AOM diagnostic criteria defined by both National and Regional guidelines include the contemporary presence of: (1) acute, recent onset of symptoms; (2) signs of inflammation in the tympanic membrane; (3) presence of middle ear effusion. There is no consensus on the type of score to be used to grade the severity of AOM, however high fever and earache not responsive to pain-relieving drugs are usually considered suggestive for severe AOM [12, 13]. Regional guidelines suggest immediate antibiotic treatment in every severe AOM, instead of national recommendations where watchful waiting strategy is considered when AOM is unilateral and occurred in children older than 2 years.

Moreover, Italian guidelines recommend immediate antibiotic therapy to those patients aged < 2 years with mild and bilateral AOM. On the contrary regional recommendations suggest observational management in children aged 6–24 months with mild unilateral or bilateral AOM (Table 1).

**Table 1:** Reports comparison between National and Emilia Romagna guidelines

	Emilia Romagna 2015		Italian Society of Paediatric 2010			
	Severe	Mild	Severe		Mild	
			Unilateral	Bilateral	Unilateral	Bilateral
Age < 6 months	ATB	ATB	ATB	ATB	ATB	ATB
Age 6–12 months	ATB	ATB/WW*	ATB	ATB	WW	ATB
Age 12–24 months	ATB	WW	ATB	ATB	WW	ATB
Age > 24 months	ATB	WW	WW	ATB	WW	WW

Comparison between Italian and regional guidelines for diagnosis and treatment of acute otitis media. ATB = immediate antibiotic. WW = watchful waiting strategy. * On the basis of paediatrician clinical judgment

Despite of science progress, AM is still a severe disease that needs parenteral antibiotics and hospitalization [14]; in fact it could lead to high morbidity and mortality due to its complications, estimated between 1.9 and 35% in different series [15–18]. Some studies reported an increased incidence of AM and its complications in the youngest children [19–21].

The aim of our study was to analyse variations in AM in children admitted to our Pediatric Emergency Department during a 15 year’s period in order to identify possible risk factors for AM and its complications.

## Methods

We performed a retrospective study of all patients aged 1 month to 14 years with AM admitted to our academic tertiary care Pediatric Emergency Unit from 1st January 2002 to 31st December 2016. Our center is an urban, academic, tertiary care Pediatric Emergency Unit consisting of a Pediatric Emergency Department with approximately 23,000 visits per year of children under 14 years. All the patients with a clinical diagnosis of AM were included. There is lack of consensus about the criteria and strategies for diagnosing AM [4]. In literature, the diagnostic criteria most commonly used include clinical signs of AOM (ongoing or within 14 days) associated with retroauricular signs of infection: swelling, erythema or tenderness over the mastoid area, antero-inferior displacement of the auricle and decrease of posterior upper wall of the auditory canal [4, 19]. According to this, in our study AM diagnosis required two or more of these signs.

We revised all the medical charts of these children and collected demographic information, patient’s history, immunization status, clinical signs and symptoms, laboratory data (white cell blood count (WBC), C-reactive protein (CRP), microbiological cultures), radiological and microbiological findings, treatment during the hospital stay and the outcome.

All of the parents gave consent to collect and analyse data contained in their children medical records.

Patients with immunodeficiencies or cranio-facial malformations were excluded from the study, since they are considered as predisposing factors for AM. Moreover, according to literature, patients with concurrent cholesteatoma or previous surgery for cholesteatoma were also excluded, since they are considered specific conditions [4, 19].

All patients with AM suspicion were visited and underwent otoscopy by both a paediatrician and an otolaryngologist and all were hospitalized. At admission we performed laboratory exam, such as WBC count and CRP level, in all patients. Cultures were obtained from the middle ear fluid after tympanocentesis or ear discharge, or from the mastoid cavity when mastoidectomy was performed. In case of tympanocentesis, the ear canal was cleaned with 70% alcohol for one minute and the fluid was removed by suction, then the tympanic membrane was punctured with a 20-gauge needle. When ear discharge was present, the middle ear fluid was directly collected from the tympanic membrane perforation after cleaning of the external ear canal with a dry cotton swab. Each specimen was collected with eSwab system, consisting of a flocked swab and a screw-capped transport tube containing 1 mL of liquid Amies medium, and inoculated into blood agar, mannitol salt agar and herellea agar. All cultures were incubated for at least 48 h at 35 °C, and resultant bacteria were identified using Gram staining and biochemical tests and underwent antibiotic sensitivity tests. Moreover we performed blood cultures and we search for urine pneumococcal antigen. According to previous studies [7, 14], we reserved imaging studies to patients with suspected AM-related complications. All patients were treated with systemic antibiotics, in agreement with our otolaryngologist, while miringotomy or mastoidectomy has been reserved only to patients who didn’t improve within 48 h.

All calculations were performed with the commercially available program IBM SPSS 20.0 for Windows (Statistical Package for Social Science, Chicago, IL). Qualitative variables were reported as absolute frequencies and percentages and were analysed using Pearson’s χ^2^ test. Quantitative data were expressed as mean values ± standard deviation and were analysed using a two – sided Student’s test. We used Mann Whitney test to compare median age between subsamples. A p – value less than 0.05 was considered as significant for each test. Comparisons were made between subgroups divided considering risk factors as prior antibiotic treatment, age less than 2 years and fever higher than 38.5 °C. Moreover we compared subsamples with or without AM complications.

## Results

A total of 149 episodes of AM occurred in 143 patients during 2002–2016 in our Pediatric ED. Of these, 2 patients were excluded due to their medical history (acute lymphoblastic leukemia and atresia auris). No one in our population had cholesteatoma. Therefore 147 AM episodes were included in the study. Information regarding vaccination against pneumococci status was available in 103 patients, 60 of which were vaccinated.

We found an increasing number of AM per year during the 15-year study, without any significant rise neither regarding patients with AOM nor considering all children admitted to our Emergency Department (Table 2). However this trend doesn’t result statistically significant (*p* = 0.06).

**Table 2:** Number of patients admitted to our Emergency Department because of AOM, AM and total of patients admitted per year

Year	Number (%) of patients with acute mastoiditis	Number (%) of patients with acute otitis media	Number of patients presenting at the Pediatric Emergency Dept.
2005	6 (0.01)	450 (0.03)	15,593
2006	12 (0.03)	451 (0.02)	18,251
2007	9 (0.02)	454 (0.02)	18,923
2008	9 (0.03)	340 (0.02)	18,922
2009	8 (0.03)	297 (0.01)	19,384
2010	8 (0.03)	274 (0.01)	19,216
2011	6 (0.02)	256 (0.01)	19,372
2012	6 (0.02)	249 (0.01)	18,884
2013	7 (0.02)	295 (0.01)	21,523
2014	13 (0.05)	251 (0.01)	22,886
2015	20 (0.07)	282 (0.01)	22,802
2016	17 (0.05)	326 (0.01)	23,197

Of 147 AM episodes, 97 (66%) occurred in boys and 50 (34%) in girls. Mean age was 4.8 ± 3.6 years, median age was 4 years (range 0.3–13.5) and 42 patients (28.2%) were younger than 2 years of age. In 136 cases a recent episode of AOM, defined as less than 2 weeks, was documented (92%) by a pediatrician. 86 patients (63%) had received an antibiotic treatment for a mean duration of 4 ± 3 days. The drug prescribed was a beta lactam in the 74% of the AOM cases and the most used was amoxicillin plus clavulanic acid (86%).

Table 3 summarizes the data of patients considered as whole group.

**Table 3:** Historical, clinical and laboratory data of the enrolled patients

	All (N 147)		All (N 147)
Mean age (years)	4.8 ± 3.6	Prior antibiotic treatment (%, n° pts)	58 (86)
Median age (years)	4 (0.3–13.5)	Complications (%, n° pts)	9 (14)
Hospitalization (days)	8.6 ± 6.8	Fever (%, n° pts)	78 (114)
Prior antibiotic treatment (days)	2.4 ± 3.0	Othalgia (%, n° pts)	77 (113)
Symptom duration (days)	5.0 ± 4.1	Othorrea (%, n° pts)	25 (37)
WBC (10^3/mmc)	15,530 ± 5350	Protrusion of auricle (%, n° pts)	95 (65)
CRP (mg/dl)	7.5 ± 10.2	Retroauricular swelling (%, n° pts)	61 (89)
Antibiotic treatment (days)	8.4 ± 4.2	Retroauricular erythema (%, n° pts)	73 (106)
Antibiotic treatment (mg/kg/die)	69 ± 20	Retroauricular pain (%, n° pts)	74 (51)

At the admission, blood culture was performed in 53% of the AM episodes and it never showed bacteraemia. 58% of these cases had received a prehospital antibiotic treatment for AOM. Cultures from the middle ear were obtained in 21 patients, in 8 cases samples came from the middle ear fluid or mastoid bone after surgery, in the remaining 13 cases they were performed from spontaneous ear discharge. 28% of cases they were positive; Pseudomonas Aeruginosas was isolated in 3 episodes, Candida species in 2 and Streptococcus Pneumoniae in 1 case. All of pathogens, except for Candida in one case, were isolated from spontaneous ear discharge. No one pathogen resulted drug resistant. We searched for pneumococcal antigen in 30 urine samples and we found it in 3 patients.

At admission for AM, all patients started parenteral antibiotics. First line therapy was ceftriaxone (69 ± 20 mg/kg/die) and it was mainly used as single therapy (93%). The mean duration of treatment was 8.4 ± 4.2 days and the majority of patients (97%) continued oral antibiotics after discharge, for a mean time of 6.5 ± 3.4 days. The first antibiotic choice at discharge was amoxicillin clavulanate (62%), followed by cephalosporins (27%) and macrolides (1%).

From the comparison between subsamples resulted that children previously treated for AOM were significantly older (5.5 ± 3.7 vs. 3.8 ± 3.1 years, *p* < 0.005) and received parenteral antibiotics for longer time (9.2 ± 4.7 vs. 7.3 ± 3.2 days, p < 0.005). Moreover, considering the subgroup of patients younger than 2 years and recently treated for AOM, they received a prior antibiotic therapy for a shorter period (1.5 ± 3.1 vs. 2.7 ± 2.9 days, *p* < 0.05). Lastly, younger patients were treated with ceftriaxone at significantly higher dosage (75 ± 17 vs. 66 ± 20 mg/kg/die, *p* < 0.01).

When we considered patients with fever, they showed significantly higher CRP (9.2 ± 11.3 vs. 2.8 ± 3.4 mg/dl, *p* < 0.0001) and WBC (16,250 ± 5405 vs. 13,470 ± 4680/mmc, *p* < 0.01) and they were treated at higher dosage (72 ± 19 vs. 59 ± 19 mg/kg/die, *p* < 0.001), furthermore they were significantly younger (4.1 ± 3.1 vs. 6.9 ± 4.1 years, *p* < 0.0001).

Table 4 compares subgroups considering as risk factors prior antibiotic treatment, age less than 2 years and fever higher than 38.5 °C.

**Table 4:** Historical, clinical and laboratory data of the subgroups considering as risk factors prior antibiotic treatment, age less than 2 years and fever higher than 38.5 °C

	Age ≤ 2 yr. (N 42)	Age > 2 yr. (N 105)	*P* value	Prior antibiotic (N 86)	No prior antibiotic (N 61)	P value	Fever (N 109)	No fever (N 38)	P value
Mean Age (years)	–	–	–	5.5 ± 3.7	3.8 ± 3.1	< 0.005	4.1 ± 3.1	6.9 ± 4.1	< 0.0001
Median Age (years)	–	–	–	4.8 (0.4–13.2)	2.7 (0.3–13.5)	< 0.005	3.3 (0.4–12-6)	6.3 (0.3–13-5)	< 0.0001
Hospitalization (days)	9 ± 3.4	8.5 ± 7.8	NS	8.7 ± 5.2	8.5 ± .8.7	NS	8.8 ± 7.1	8.1 ± 5.8	NS
Prior antibiotic treatment (days)	1.5 ± 3.1	2.7 ± 2.9	< 0.05	–	–		2.3 ± 3.1	2.6 ± .2.9	NS
Symptom duration (days)	4.2 ± 3.9	5.3 ± 4.2	NS	6 ± 4.4	3.7 ± 3.2	< 0.0001	5.2 ± 4.3	4.4 ± 3.4	NS
WBC (10^3/mmc)	18,440 ± 6115	14,370 ± 4550	< 0.0001	14,550± 5090	16,920± 5450	< 0.01	16,250 ± 5405	13,470 ± 4680	< 0.01
CPR (mg/dl)	9.5 ± 7.7	6.8 ± 11	NS	6.1 ± 5.9	9.6 ± 14.1	NS	9.2 ± 11.3	2.8 ± 3.4	< 0.0001
Antibiotic treatment (days)	8.9 ± 3.4	8.2 ± 4.5	NS	9.2 ± 4.7	7.3 ± 3.2	< 0.005	8.6 ± 3.6	8 ± 5.2	NS
Antibiotic treatment (mg/kg/die)	75 ± 17	66 ± 20	< 0.01	69 ± 20	69 ± 19	NS	72 ± 19	59 ± 19	< 0.001
Complications (%, n° pts)	14 (6)	8 (8)	NS	65 (9)	35 (5)	NS	10 (11)	8 (3)	NS
Fever (%, n° pts)	86 (36)	69 (73)	< 0.05	74 (64)	82 (50)	NS	–	–	–
Othalgia (%, n° pts)	48 (20)	88 (93)	< 0.0001	78 (67)	75 (46)	NS	73 (83)	91 (30)	< 0.05
Othorrea (%, n° pts)	26 (11)	25 (26)	NS	30 (26)	18 (11)	NS	26 (28)	24 (9)	NS
Protrusion of auricle (%, n° pts)	76 (32)	60 (63)	NS	62 (53)	69 (42)	NS	67 (76)	59 (19)	NS
Retroauricular swelling (%, n° pts)	78 (33)	54 (56)	< 0.01	54 (63)	57 (35)	NS	65 (74)	47 (15)	NS
Retroauricular erythema (%, n° pts)	74 (31)	72 (75)	NS	70 (60)	75 (46)	NS	73 (83)	72 (23)	NS
Retroauricular pain (%, n° pts)	28 (12)	60 (62)	< 0.001	46 (54)	28 (46)	NS	46 (52)	69 (22)	< 0.05

Historical, clinical and laboratory data of the subgroups considering as risk factors prior antibiotic treatment, age less than 2 years and fever higher than 38.5 °C. NS = not significance

Fever (86% vs. 69%, *p* < 0.05) and retroauricular swelling (78% vs. 54%, p < 0.01) were significantly more common in the younger children, 0–2 years. On the other hand, older patients reported more easily earache (88% vs. 48%, p < 0.0001) and retroauricular pain (60% vs. 28%, p < 0.001). Accordingly, feverish children - who are younger than non-feverish ones - presented less frequently earache (73% vs. 91%, p < 0.05) and retroauricular pain (46% vs. 69%, p < 0.05).

At least one complication was diagnosed in 14 cases (9.5%), the majority of which was male (11/14). Patients with AM complications had a mean age of 3.4 ± 2.5 years. The type of complications and their incidence are listed in Table 5. Subperiosteal abscess was the most common complication (37%) in our population. No patient died.

We compared complicated AM cases with non-complicated ones and we didn’t find any risk factors of complications (Table 6). Retroauricular erythema was the only clinical sign inversely related to AM complications. In fact non – complicated cases presented more frequently this peculiarity (76% vs 36%, *p* < 0.01).

**Table 5:** Complications associated to acute mastoiditis in our population

	N°	%
Subperiosteal Abscess (SPA)	6	37
Labyrinthitis	2	13
Venus sinus thrombosis (VST)	2	13
Bezold Abscess	1	6
Gradenigo Syndrome	1	6
Facial nerve palsy	2	13
Horner Syndrome	1	6
XII nerve palsy	1	6

**Table 6:** Historical, clinical and laboratory data and comparison between patients with or without complications

	Complicated (N 14)	Not complicated (N 133)	P – value		Complicated (N 14)	Not complicated (N 133)	P – value
Mean Age (years)	3.4 ± 2.5	4.9 ± 3.6	NS	Fever (%, n° pts)	78 (11)	74 (98)	NS
Median age (years)	3 (0.8–10)	4.1 (0.3–13.5)	NS	Othalgia (%, n° pts)	50 (7)	80 (106)	NS
Hospitalization (days)	16.8 ± 8.1	7.7 ± 6.1	< 0.001	Othorrea (%, n° pts)	78 (11)	57 (98)	NS
Prior antibiotic therapy (days)	2.7 ± 2.8	2.8 ± 3.1	NS	Protrusion of auricle (%, n° pts)	43 (6)	67 (89)	NS
Symptom duration (days)	7 ± 3.5	4.8 ± 4.1	< 0.05	Retroauricular swelling (%, n° pts)	50 (7)	62 (82)	NS
WBC (10^3/mmc)	17,250 ± 8110	15,350 ± 4990	NS	Retroauricular erythema (%, n° pts)	36 (5)	76 (101)	< 0.01
CRP (mg/dl)	8.1 ± 8.1	7.5 ± 10.5	NS	Retroauricular pain (%, n° pts)	36 (5)	52 (69)	NS
Antibiotic treatment (days)	15 ± 9	7.7 ± 2.6	< 0.01	Antibiotic treatment (mg/kg/die)	88.7 ± 19.8	67.5 ± 19.5	< 0.01

Historical, clinical and laboratory data and comparison between patients with or without complications. NS = not significance

As foreseeable, complicated patients were hospitalized and treated with parenteral antibiotic for a longer period (*p* < 0.001, p < 0.01) and at higher dosage (p < 0.01). Moreover complicated patients referred previous symptoms for a longer time (*p* < 0.05).

We performed radiological imaging in case of complications’ suspicion, in fact in our population there is a strict predicable connection between complications, imaging and surgery (*p* < 0.0001, p < 0.0001). Imaging was made in 32 cases: 25 cranial computed tomography, 3 magnetic resonance and in 4 cases we performed both. In 3 cases imaging revealed coalescent mastoiditis, 6 patients had subperiosteal abscess and in 2 cases we found venous sinus thrombosis.

Ten cases (6.8%) underwent surgery. Myringotomy was performed in 8 patients, in 3 cases it was the only procedure, while in 5 cases it was associated to mastoidectomy. Three patients of this subgroup underwent also to a tympanostomy tube placement. In 2 patients isolated mastoidectomy was performed.

## Discussion

We performed a 15-year retrospective study regarding children admitted to our Paediatric Emergency Department due to AM. This study provides updated information on demographics data, clinical presentation and management of AM in children from 2002 to 2016.

There are many controversies regarding the incidence of AM in the last decades. Some authors found an increase of AM incidence in the last decades [2, 8, 20, 22, 23]. They referred this event both to the selection of bacterial strains resistant to antibiotics [2, 8] and to the implementation of guidelines restricting the use of antibiotics in AOM [22]. On the contrary, other authors reported no changes in the overall number of cases of AM, despite the decrease in the antibiotic use for AOM [17, 18]. Groth et al. collected 577 AM cases from 34 Swedish Hospital during 1993–2007 and they didn’t find an increase in AM incidence [19]. According to these results, Anthonsen and colleagues performed a 10–year retrospective multicentre study in Denmark and found a stable incidence of AM [18]. We found an increasing number of subjects affected by AM during the last three years, albeit this tendency doesn’t result statistically significant. Moreover it is possible that our finding does not reflect the real pediatric AM incidence in Italy, even if it is consistent with another Italian multicentre trial including 25 hospitals [5].

There aren’t universally accepted guidelines for AM approach, but local algorithmic management. Many authors consider intravenous antibiotics sufficient to successfully treat most children with AM [3, 15], while others support the importance of routine miringotomy in order to relief from pain and prevent complications [7, 22]. In our population, we used to treat uncomplicated AM only with systemic antibiotics, in agreement with the ENT specialist, while miringotomy or mastoidectomy were reserved to patients who didn’t improve to antibiotic treatment within 48 h.

There is not a standard medical treatment for pediatric AM, however empiric antimicrobial therapy should provide coverage for the principal pathogens: S. pnuemoniae[3, 16], S. pyogenes and *S. aureus* (including methicillin resistant *S. aureus* in high prevalence areas). Therefore intravenous beta lactamase antibiotics are usually used as first line treatment, mostly as monotherapy [5, 15, 20, 24, 25], but also associated to amino glycosides [3, 26], clindamycin [7], fosfomycin [27]. According to literature [5], we used parenteral Ceftriaxone considering the low prevalence of MRSA in our population.

The positive outcome in our population supports this choice even if we have not meaningful microbiological data. In fact we isolated Streptococcus Pneumoniae in middle ear fluid only in 1/21 cultural exam and we found pneumococcal antigen in only 3/30 urine samples. Regarding other pathogens isolated, Candida species and Pseudomonas aeruginosa, we supposed that they could represent a contamination from the ear canal. In particular, Pseudomonas aeuriginosas was isolated in 3 patients from spontaneous ear discharge. Since it represents the most common pathogen for chronic suppurative AOM and the most common cause of external otitis, we suppose that it could represent a contamination from the ear canal.

As there is a better coverage in PCV immunization in our population over this 15 years period, we speculate that the rise in AM incidence could be due to the infection by pneumococcal serotypes not included in current vaccines or to pathogen other than Streptococcus Pneumoniae. A recent study showed that, since introduction of PCV7, serotype 19A emerged as principal pathogen within children with pneumococcal mastoiditis. This serotype resulted highly resistant to penicillin and macrolides and its domain remained stable even after introduction of PCV13 despite it was encompassed in this vaccine [1]. In Italy 7-valent pneumococcal conjugate vaccine (PCV7) was available since 2002 and it was added to National Immunization Program in 2006 in 21 regions. From 2010 PCV7 has been replaced by 13-valent vaccine (PCV13), which was finally inserted in the National Immunization Program 2012–2014.

In our population we knew the PCV immunization status of only 103/143 enrolled children with AM. Therefore we haven’t enough data to draw conclusions about the correlation between the rise in AM incidence and the PCV coverage. Another reason that may justify this finding is the possible increase of antibiotic resistance. We can stand that outdoor patients weren’t treated accordingly to AOM guidelines in the majority of cases; in fact amoxicillin plus clavulanic acid was the first choice for AOM therapy, instead of amoxicillin alone. The abuse of amoxicillin plus clavulanic acid might have influenced the rise of resistant pathogens, however we don’t have enough microbiological data to correctly interpret changes in pathogens and antibiotic resistance. Finally in most children with AM the disease arose after an initial AOM episode, thus the “watchful waiting strategy” recommended by the latest AOM national and regional guidelines [5, 13] may have influenced trends in paediatrics mastoiditis.

The increasing trend of AM evidenced in our population in the last decade should be cautiously taken in consideration, in fact our data are limited to a single medical center and we don’t have enough information regarding microbiological isolation or AOM episode, to identify the real reason for this variation.

Many authors reported a higher incidence of AM and its complications in younger children [19, 25, 28]. Our experience did not confirm these findings. We reported that patients older than 2 years were more frequently treated for AOM than the younger ones; moreover they referred more often AM symptoms than pre-verbal patients, due to the low ability of the toddlers to express earache or retroauricular pain. Therefore we expected that younger children may have presented a faster progression from AOM to mastoiditis or that AOM may have been misunderstood due to low ability to report symptoms. Nevertheless, we found no differences in incidence of AM and its complications between patients younger and older than 2 years. One possible explanation of our results is the variation in antibiotic dosage in AM emerged from our study. Children younger than 2 years were treated at higher dosage for mastoiditis, probably because they were more frequently feverish and they had higher levels of CRP and WBC. Since we considered fever and age < 2 years as risk factors for AM complications, we hypothesize that these risk factors had not impacted on complications’ rate due to higher antibiotics dosage in this group.

Complications’ rate reported in literature ranges from 1.9 to 35% [17, 18]. According to this data, we reported 9.5% of complicated cases, mainly represented by subperiosteal abscess. We performed radiological imaging only in case of complications’ suspicion to reduce radiation exposure and anaesthetic risk. In literature the use of radiological imaging is controversial in pediatric AM. Some authors recommend diagnostic imaging at presentation in order to confirm diagnosis and to rule out complications [24, 29]. However, as reported in literature, the outcome of treatment in series using imaging doesn’t seem to be better than series avoiding these exams. Therefore imaging could be employed only in case of prolonged or complicated course of AM [14, 18, 30].

We didn’t identify any risk factors for AM complications however we found a significant inverse relationship between retroauricular erythema and complications. We speculate that this sign may have permitted a more precocious diagnosis of AM and, therefore, a more immediate treatment.

Our study has some limitations due to its retrospective nature; in fact there was no standardization in medical charts and some findings were missing, in particular regarding historic data. Moreover we cannot locate those patients with AOM in which “watchful waiting strategy” was practiced.

## Conclusion

According with previous studies, the incidence of AM in our population has not been reduced during the last years despite pneumococcal vaccine’s introduction. We have to face all the reasons why this condition is still relevant, such as antibiotic resistance, new pathogens involved and a possible role played by the implementations of national and international therapeutic AOM guidelines restricting the use of antibiotics in this disease. Moreover it is mandatory to improve the management of AOM in order to prevent mastoiditis. In our local reality there is an important discrepancy between paediatrician behaviour and AOM guidelines. An early diagnosis and treatment of AM is essential to prevent serious complications. We suggest greater attentions to signs and symptoms in pre-verbal children, when AOM or AM could be misdiagnosed and appropriate treatment may be delayed.

Future studies are needed in order to confirm these trends and to better clarify risk factor for AM development.
